# In Situ Imaging during Compression of Plastic Bonded Explosives for Damage Modeling

**DOI:** 10.3390/ma10060638

**Published:** 2017-06-10

**Authors:** Virginia W. Manner, John D. Yeager, Brian M. Patterson, David J. Walters, Jamie A. Stull, Nikolaus L. Cordes, Darby J. Luscher, Kevin C. Henderson, Andrew M. Schmalzer, Bryce C. Tappan

**Affiliations:** 1 Explosive Science and Shock Physics Division, Los Alamos National Laboratory, Los Alamos, NM 87545, USA; vwmanner@lanl.gov (V.W.M.); jamie.stull@lanl.gov (J.A.S.); aschmalz@lanl.gov (A.M.S.); btappan@lanl.gov (B.C.T.); 2 Materials Science and Technology Division, Los Alamos National Laboratory, Los Alamos, NM 87545, USA; bpatterson@lanl.gov (B.M.P.); ncordes@lanl.gov (N.L.C.); kch@lanl.gov (K.C.H.); 3 Theoretical Division, Los Alamos National Laboratory, Los Alamos, NM 87545, USA; djwalters@lanl.gov (D.J.W.); djl@lanl.gov (D.J.L.)

**Keywords:** X-ray computed tomography, mesoscale modelling, explosives, polymer-matrix composites

## Abstract

The microstructure of plastic bonded explosives (PBXs) is known to influence behavior during mechanical deformation, but characterizing the microstructure can be challenging. For example, the explosive crystals and binder in formulations such as PBX 9501 do not have sufficient X-ray contrast to obtain three-dimensional data by in situ, absorption contrast imaging. To address this difficulty, we have formulated a series of PBXs using octahydro-1,3,5,7-tetranitro-1,3,5,7-tetrazocine (HMX) crystals and low-density binder systems. The binders were hydroxyl-terminated polybutadiene (HTPB) or glycidyl azide polymer (GAP) cured with a commercial blend of acrylic monomers/oligomers. The binder density is approximately half of the HMX, allowing for excellent contrast using in situ X-ray computed tomography (CT) imaging. The samples were imaged during unaxial compression using micro-scale CT in an interrupted in situ modality. The rigidity of the binder was observed to significantly influence fracture, crystal-binder delamination, and flow. Additionally, 2D slices from the segmented 3D images were meshed for finite element simulation of the mesoscale response. At low stiffness, the binder and crystal do not delaminate and the crystals move with the material flow; at high stiffness, marked delamination is noted between the crystals and the binder, leading to very different mechanical properties. Initial model results exhibit qualitatively similar delamination.

## 1. Introduction

Octahydro-1,3,5,7-tetranitro-1,3,5,7-tetrazocine (HMX) is a powerful high explosive that is routinely used in a variety of plastic bonded explosive (PBX) formulations such as PBX 9501. Many HMX-based PBXs are highly loaded (10% or less binder) and exhibit bimodal or trimodal particle size distribution in the size range of single to hundreds of micrometers. In addition to safety considerations, PBXs are difficult to characterize from a materials science standpoint because they are relatively fragile and often have low melting points, poor optical or X-ray contrast between the crystals and the binder, and extensive intermixing of constituents. However, the microstructure of PBX materials is known to directly influence fracture behavior [1,2], mechanical properties [3,4], and thermal properties [5,6]. Microstructural behavior is also believed to strongly affect the initiation process, particularly in off-normal scenarios such as cook-off or oblique impact/skid testing [7,8]. For example, microstructure-based effects such as binder thermal expansion and ventilation pathways through the material have been shown to influence time-to-ignition in cook-off of PBX 9501 [9]. Advancing our current understanding of these links between the PBX microstructure and properties is hindered by the difficulty of characterizing the PBX at the mesoscale and would be greatly benefited by systematic, mesoscale experiments coupled with robust model development.

PBX 9501 is widely studied due to its use in a variety of critical applications, and has an extensive amount of historical experimentation data [10,11,12,13]. Mesoscale model development is critical to understanding this treasure trove of data. Currently, models are hindered by a lack of precise microstructural characterization, particularly for in situ measurements during mechanical deformation. Critical information such as crystal-binder adhesion, crystal movement during deformation, and void or crack nucleation and growth are generally only known (if at all) by measurements on bulk specimens [14], by surface-only measurements [15], or by post-test characterization [16]. Three-dimensional microstructural data can be acquired during deformation by in situ imaging such as X-ray computed tomography (CT), but the HMX and the polymer binder have similar density and the X-ray contrast is not sufficient to segment the two phases from each other.

To address this difficulty, we have formulated a series of HMX-based PBXs using lower-density binders which will allow for microCT characterization. Specifically, we have prepared formulations with 88% HMX using hydroxyl-terminated polybutadiene (HTPB) binder in order to form an explosive that is relatively insensitive to mild stimuli, analogous to PBXN-110 [17], differing primarily in the use of dioctyladipate (DOA) as a plasticizer. A particle size distribution of 75% coarse Class 1 HMX and 25% fine Class 2 HMX was used here, which is identical to the distribution found in PBX 9501 [18]. Small changes were made to the mechanical stiffness of the binder system in order to determine how rigidity plays a role in damage of the composite explosive. The HMX content here is much lower than PBX 9501 (95% HMX) and so a direct comparison between the materials must be done with caution, but the characterized microstructure should still provide insight into general PBX damage mechanisms and the relative effects of binder strength and crystal-binder adhesion. Although microCT imaging during compression has been performed in similar non-explosive materials [19,20], this is the first methodical study we are aware of to directly demonstrate the importance of the binder system using explosive samples.

## 2. Materials and Methods 

Coarse HMX (Class I) and fine HMX (Class II) were produced by Holston Army Ammunition Plant (HAAP, Kingsport, TN, USA). MDI/Isonate (diphenylmethane diisocyante; Isonate 143-L MDI) and lecithin were obtained from Firefox Enterprises (Pocatello, ID, USA), and dibutyl tin dilaurate from TCI America (Portland, OR, USA). HMX, HTPB, and DOA were dried overnight in a vacuum oven at 60 °C. 

**Figure 1:**
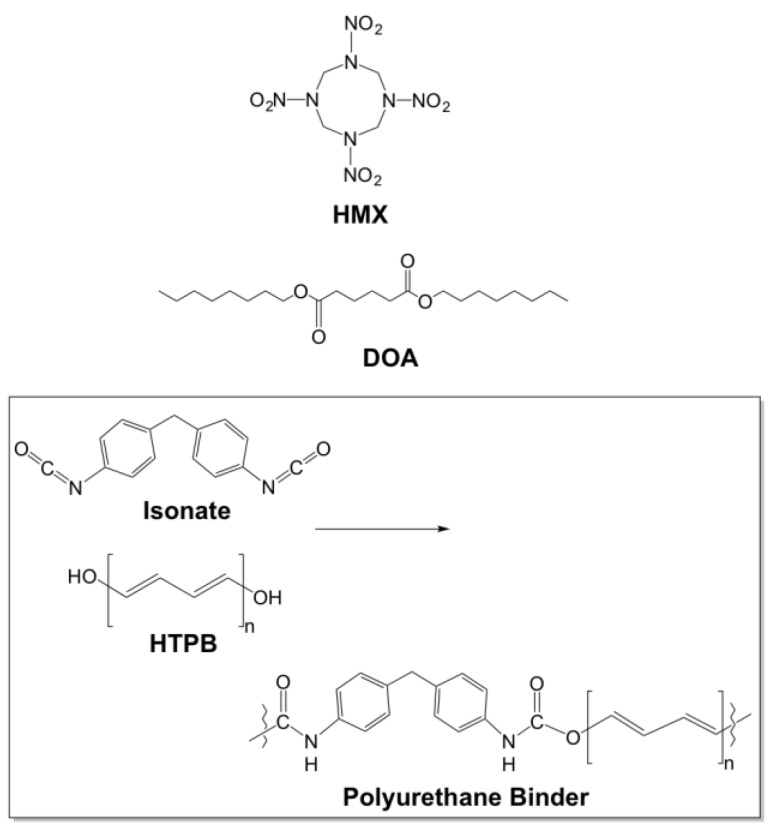
Chemical components of the HMX/HTPB formulation, where HTPB cures with isonate to form the polyurethane binder system surrounding the HMX crystals and DOA performs as a plasticizer.

Four formulations were prepared and tested in this work. Two formulations were comprised of 88% HMX, 5.4% HTPB, 5.4% DOA, 0.7% lecithin, and trace dibutyltin dilaurate as a catalyst. Figure 1 shows the chemical structures of these formulations. Two different levels of diphenylmethane diisocyanate (isonate) were used to cure the HTPB and form a polyurethane binder system surrounding the HMX crystals. Here, these are simply labeled “**L**” (low isonate; 0.5%) and “**H**” (high isonate; 1%). 

The other two formulations used HMX bound with various mixtures of glycidyl azide polymer (GAP) cured with a commercial blend of acrylic monomers/oligomers (**AMO**) capable of cycloaddition with the azide functionality on the GAP. The four formulations are shown in Table 1. Both binder systems are cast-curable, which offers greater flexibility in the preparation of samples than the traditional slurry-formulated materials. This flexibility enabled simple but precise variations in the mechanical properties of the binders in order to study the resulting effects in the composite. However, one drawback is that sample preparation with highly uniform dimensions can be difficult, a result which is discussed in the next section. 

To prepare **CF75H**, fine HMX (1.1 g) was mashed with a Teflon plunger to remove clumps, and mixed well with course HMX (3.3 g). HTPB (0.27 g), DOA (0.27 g), lecithin (0.035 g), MDI/Isonate (0.051 g), and trace dibutyltin dilaurate were mixed together well in an aluminum pan. The HMX was then added to the liquid mixture, and mixed with a spatula and gloved hands to ensure that the formulation was well mixed. The mixture was placed in a small plastic container and alternated through vacuum mixing and a centrifuge. The formulation was allowed to cure overnight. **CF75L** was prepared using the same method, but with 0.027 g isonate to reduce the rigidity of the binder system. See Table 1 for variations in the amount of isonate and ratio of HMX coarse/fine. After curing, small ~5 mm diameter cylinders were prepared using brass cork borers.

**CF75-AMO1** and **CF75-AMO2** were hand mixed on a 2 g scale using the quantities listed in Table 1. After mixing, the materials were packed well into 5–7 mm diameter straws, irradiated with UV-light, and allowed to cure overnight before cutting open the straws. They were cut into 7–10 mm lengths using a blade to shave the ends to be as flat as possible.

Uniaxial compression tests were conducted remotely on an Instron 8862 (Instron Corp., Norwood, MA, USA). Individual samples were prepared from the formulations by boring cylinders approximately 7 mm diameter × 8–11 mm height. Tests were conducted in position control and the strain was measured using the displacement of the crossheads of the instrument. All specimens were tested at ambient conditions and the crosshead speed was varied around the 0.1 in/min value according to the actual specimen height to give a strain rate for all tests of 0.0045 in/in/s. 

Micro X-ray CT images were collected using an Xradia Carl Zeiss X-ray Microscopy Inc. MicroXCT (Pleasanton, CA, USA). The system uses a microfocused X-ray source with a tungsten anode. The source was operated at 40 kilovolts potential (kVp) and 10 W·A scintillator converts the X-ray photons to visible light photons and is mounted on the front of the 4× magnification objective which then transmitted the optical photons to the 1 k × 1 k piezo electrically cooled charge-coupled device (CCD). With an individual exposure time of 20 s, the 2001 images were collected as the sample was rotated 180°. These conditions produced an isotropic voxel size of 5.2 micrometers at the sample. The images were reconstructed using TXM reconstructor (Carl Zeiss X-ray Microscopy Inc., Pleasanton, CA, USA) and rendered and quantified using Avizo (FEI, Hillsboro, Oregon, USA). The images were smoothed with an edge-preserving filter and segmented based upon the gray-scale values and the crystals, binder, and voids were separated and quantified. In situ CT imaging of the composites during loading was completed using a Deben CT500 500N loadcell (Deben UK LTD., London, UK).

## 3. Results and Discussion

### 3.1. Mechanical Testing of HMX Formulations

Stress-strain data was collected for each formulation in the uniaxial compression configuration. The **CF75L** specimens had an average density of 1.566 ± 0.067 g/cm^3^, **CF75H** specimens had a density of 1.572 ± 0.148 g/cm^3^, the **CF75-AMO1** specimens had a density of 1.649 ± 0.086 g/cm^3^, and the **CF75-AMO2** specimens had a density of 1.680 ± 0.086 g/cm^3^. 

**Figure 2:**
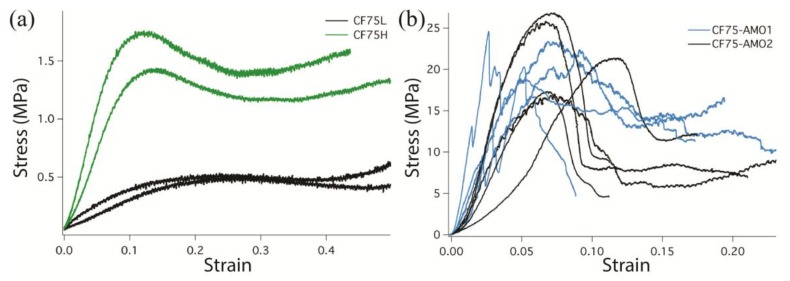
Unaxial compression stress-strain curves for the HTPB binder formulations **CF75L** and **CF75H** (**a**) and the **CF75-AMO** formulations (**b**). Note the different scales—the HTPB samples in (**a**) are more compliant. Compression tests were performed at ambient temperature and a strain rate of 0.0045 in/in/s.

Figure 2 shows the compression stress-strain data for all four specimens. In general the **CF75L** and **CF75H** specimens have much lower strength than the **CF75-AMO** specimens. The **CF75L** specimens exhibited low strength and significant plastic flow during uniaxial compression; only at the very end of the tests did the edges of the material start to tear. The **CF75H** specimens registered higher strength than the **CF75L** specimens, which is expected due to the higher isonate content. The **CF75H** specimens also deformed elastically compared to the **CF75L** specimens that failed plastically, with little to no recovery observed after the load has been removed. In contrast, the **CF75-AMO** specimens exhibited very brittle failures that were not observed for the HTPB-bonded formulations. These specimens had much higher compressive strength and more extensive fracture compared to the softer **CF75L** and **CF75H** materials during uniaxial compression. 

The **CF75-AMO** samples did not have good reproducibility so additional samples were tested, five samples in total for each. It is clear from Figure 2b that the stronger more brittle **CF75-AMO** materials are more sensitive to the geometry of the cylinders. Since the samples were not precisely machined, the uneven surfaces lead to larger spread in the compression data. In some cases this leads to point loading on the sample, which is observed as sharp peaks in the data, or what appears to be a change in the modulus. Here it is clear that making conclusive statements about the differences between the **AMO** samples is not possible due to the range of responses. The general behavior between them is similar.

**Figure 3:**
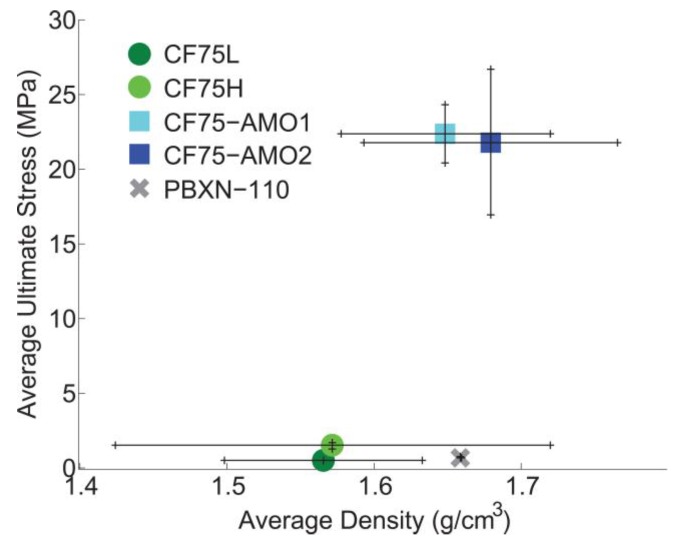
Ultimate compressive stress as a function of density for the four formulations, compared to PBX 9501 and PBXN-110.

The maximum or ultimate stresses of the specimens are plotted as a function of density in Figure 3 and compared to PBXN-110 [17]. The error bars represent the standard deviation measured for the density and the ultimate stress reached for each formulation. The compression strength of the **CF75L** and **CF75H** materials compare well to PBXN-110 although the density of PBXN-110 is closer to the **CF75-AMO** specimens. 

### 3.2. In-Situ Tomography during Compression

Samples of each formulation were sequentially 3D imaged using CT with an in situ load stage during compression. Each sample was compressed to identical engineering strain (10% and 20% displacement of the total sample height, or ε = 0.1 and ε = 0.2). They were held for 10−15 min after compression to allow any residual plastic flow to occur, then imaged at this strain [21,22]. Each full 3D measurement required approximately 26 h. 

**Figure 4:**
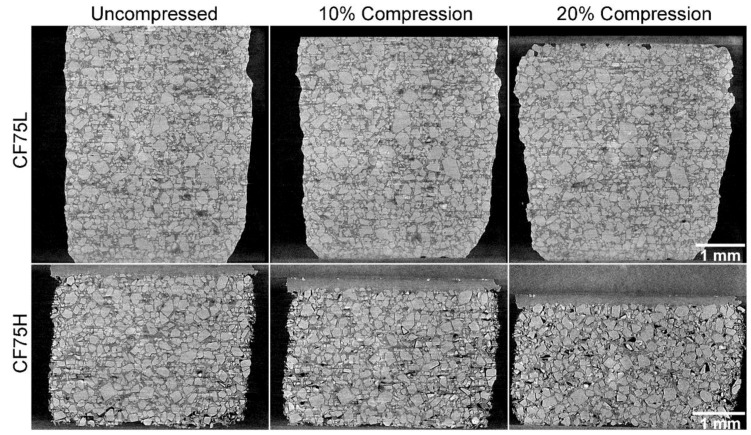
CT images of **CF75L** and **CF75H** samples under uniaxial compression. Note that **CF75L** does not show cracking, but mostly flow. **CF75H** shows a separation of the binder from the crystals.

The **CF75L** sample was found to be quite ductile and to exhibit a strong plastic flow during uniaxial compression, leading to a large Poisson effect but little cracking or separation of the binder from the crystals. However, **CF75H** was much more rigid and with delamination between the binder and crystals widely seen. The separation between the crystals and binder is not uniform throughout the cylinders. Reconstructed slices out of the full 3D data set for **CF75L** and **CF75H** at 0%, 10%, and 20% strains are shown in Figure 4.

**Figure 5:**
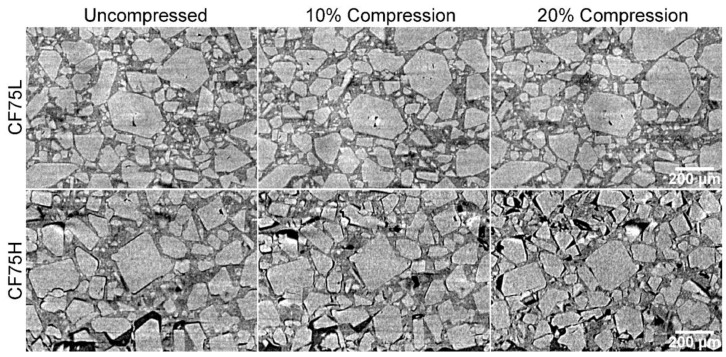
Selected regions of **CF75L** and **CF75H** from Figure 4.

The effect of increased curing via increased isonate is evident in the difference in deformation behavior between samples **CF75L** and **CF75H**. A selected region from Figure 4 is highlighted across several compression strains in Figure 5 for both materials. Individual crystals can be tracked during compression, showing increased separation from each other as the material deforms. The images were taken at the same location in the experiment reference frame (i.e., same physical x-y-z coordinates inside the instrument) but the material deforms slightly non-uniformly and so individual crystals move in or out of the single reconstructed slice during the compression. However, most of the crystals can be found in the majority of the images and this is sufficient to observe material motion, rotation of crystals, crystal damage, and void and/or crack nucleation and growth. In **CF75L** no fracture is evident even up to 20% strain, and the only changes in the microstructure are due to material motion. The binder is either soft or adhesive enough to the crystals to flow with them as they respond to the compressive stress. However, **CF75H** exhibits extensive crack formation at the crystal-binder interfaces. At 20% strain, most of the crystals have at least one surface debonded from the HTPB matrix.

**Figure 6:**
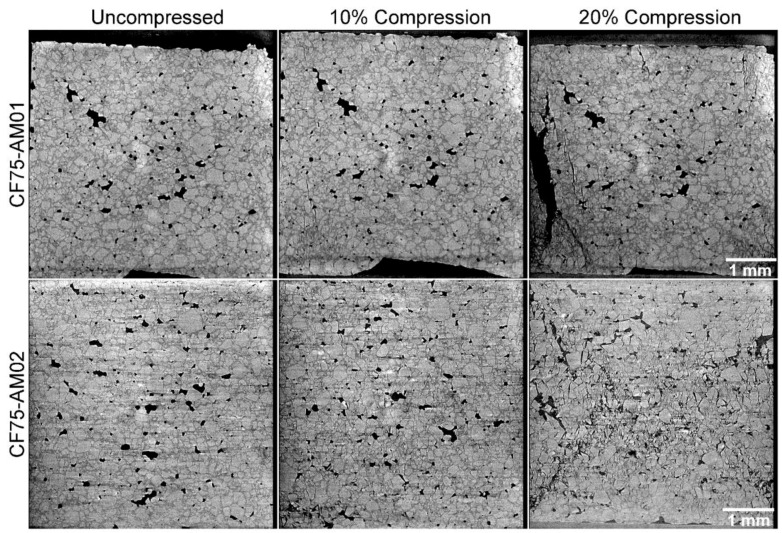
Comparison of **CF75-AMO1** and **CF75-AMO2** at increasing compressions. Both samples show extensive cracking by 20% compression, with **CF75-AMO2** exhibiting the most damage.

**Figure 7:**
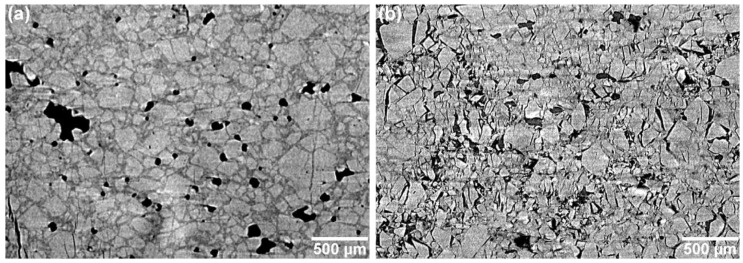
Selected regions of compressed **CF75-AMO1** (**a**) and **CF75-AMO2** (**b**) at 20% strain.

Both of the **CF75-AMO** binder specimens show increased adhesion between the crystals and the binder. Due to the higher density of the **CF75-AMO** mixtures compared to HTPB, the contrast between the HMX and the binder is diminished. However, some trends are readily apparent. Figure 6 shows compression of **CF75-AMO1** and **CF75-AMO2**. In **CF75-AMO2**, an “X” pattern of fracture is apparent in the reconstructed slice, demonstrating a conical failure which is typical of brittle materials. In **CF75-AMO1**, cracks form and extend primarily along the direction of compression in a transgranular fashion. These observations are more easily concluded via Figure 7, which highlights a selected area of the microstructure within the 20% strain tomogram for each material. The “X” pattern can be seen primarily along ~45° directions relative to the compression direction and is formed by crystals fracturing (transgranular) and delaminating from the binder (intergranular).

One particularly useful observation from the CT is that the microstructural response of the two AMO specimens are completely different, but the overall mechanical properties (cf. Figure 2) are similar. This indicates that changing the ratio of GAP and AMO more strongly affects the interfacial adhesion between HMX and the binder than the stiffness of the binder. Both effects are interlinked but the change from intergranular (**CF75-AMO1**) to mixed inter- and transgranular (**CF75-AMO2**) failure demonstrates a clear change in adhesive properties. Further quantification is beyond the scope of the present study but could help explain large differences in sensitivity or explosive properties in other PBXs which otherwise have similar mechanical properties [23]. 

### 3.3 Automated Microstructural Description from the CT Data

**Figure 8:**
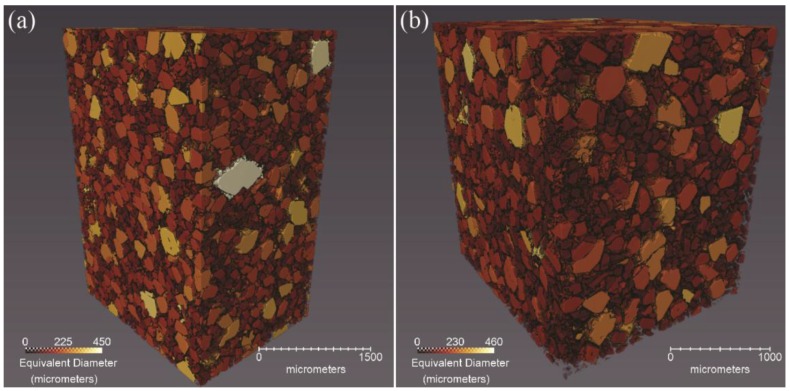
Volume rendering of **CF75L** (**a**) and **CF75H** (**b**), showing the HMX crystals colored by equivalent diameters. Larger crystals are lighter in color.

For **CF75L** and **CF75H**, excellent X-ray contrast was seen between the HTPB, the HMX crystal, and voids, allowing for image analysis software to clearly segment each constituent and therefore quantify measurements of each. Table 2 gives the percent volume and mass for the HMX crystals, binder, and voids in each of the samples as calculated by the segmentation routine. Note that the calculated HMX content is slightly below the measured formulation mass percentage. This is likely due to the moderate amount of HMX crystals smaller than 10 microns, which is approximately the resolution of the CT instrument. However, the fact that the majority of the HMX can be automatically segmented is important, as most X-ray tomography studies of PBX materials have given poor contrast between the crystals and binder to date. As a result of this, it is possible to create 3D renderings of the crystals in which each crystal is colored by its equivalent diameter (Figure 8). The equivalent diameter measurement assumes each object is a sphere and calculates its diameter. Additionally, it is possible (not shown) to measure the changes in crystal and void sizes as a function of the strain [24]. The dataset is sufficient to track the debonding behavior of a large single crystal within the matrix as a function of compression. **CF75-AMO1** and **CF75-AMO2** also give reasonable contrast between the crystals and binder by eye but the absolute contrast was not sufficient for the software routines to segment them. Note that this weaker contrast is why the microstructure in Figure 6 is less apparent than in Figure 4.

The quality of the contrast in the **CF75L** and **CF75H** systems is such that digital image correlation (DIC) could be used in future efforts to map the local strain fields, as in Croom et al. [25,26]. This could also be used to track the rotation of the crystals within the binder. Some of the larger crystals visible in Figure 5 appear to be rotating, not just translating, under the applied compressive load. This type of problem has been treated recently by Yang et al. [27] who established a theoretical framework to predict arbitrarily shaped particles rotating within a soft matrix under a remote stress. Such a theoretical approach could be linked with the particle imaging capability shown here to validate simulations of material deformation.

### 3.4. Application to Mesoscale Modeling

This type of data is critical to constructing and simulating real three-dimensional microstructures for mesoscale modeling. A primary objective for this mesoscale modeling effort is to simulate quasistatic compression of PBX 9501 specimens, which have even less contrast between the HMX and the binder than the **CF75-AMO** samples due to the higher density of the binder. However, by substituting in a low-density binder such as HTPB, while retaining an HMX particle size distribution representative of PBX 9501, a realistic, 3D, voxelized microstructure can be measured with microCT and then meshed for a finite element simulation. Additionally, utilizing a close representation of the actual microstructural geometry rather than an idealized statistical representation (i.e., random spheres, circles, etc.) is a critical component in simulating realistic mechanical responses [28]. Ideally, the simulation parameters would be validated by direct comparison to force-displacement data and the well-characterized microstructure, e.g., Figure 4. The simulation could then be applied to PBX 9501 by duplicating the initial microstructure from the HTPB samples while altering the binder properties to match those of the PBX 9501 binder.

While it is recognized that a three-dimensional (3D) description of the material geometry and associated deformation and stress states within the microstructure are critical for accurate quantitative descriptions of damage evolution in explosive materials, there is much utility in two-dimensional (2D) simulations towards that ultimate goal. For example, Arora et al. have conducted 2D and 3D simulations of explosives that explicitly include microstructure geometry [29]. While their 3D simulations employed only an idealized representation of the actual geometry (e.g., spherical particles), their 2D simulations clearly demonstrated the importance of accurately representing the actual crystallite geometry. Barua et al. have developed a cohesive finite element modeling (CFEM) approach based on 2D plane strain simulation of microstructural volume elements of polymer bonded explosives and demonstrated its utility within a framework to investigate the spatiotemporal distribution of hotspot formation during dynamic loading conditions [30,31,32]. While their simulations included an accurate 2D description of microstructure geometry, it did not include anisotropic thermoelastic behavior of the explosive crystals which may be important for capturing local stress distributions that drive the decohesion of the crystal and binder. Hu et al. have used 2D simulations to evaluate the debonding of HTPB binder from ammonium perchlorate (AP) particles and compared them with corresponding experiments to validate a CFEM approach [33]. Gonthier’s group has developed a coupled FEM-discrete element modeling (FEM-DEM) approach to identify energy dissipation and hotspot generation in microstructure simulations that are used to inform macroscale models of explosive response [34,35,36]. The 2D modeling employed in this research is a pre-cursor to guide the judicious development and use of 3D models. By assessing the consistency between qualitative trends from such 2D simulations and the truly 3D experiments, this research will narrow the scope for continued 2D and 3D model development towards predictive capabilities of damage initiation and progression to failure in PBXs.

**Figure 9:**
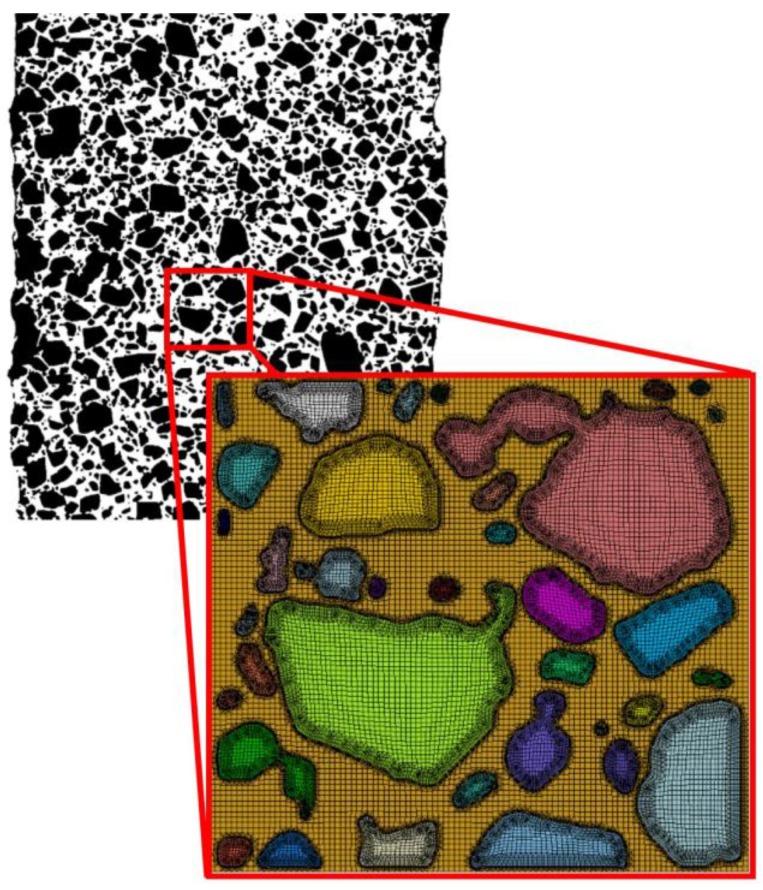
Binary image of **CF75L** with inset detail of the element mesh used in preliminary simulations.

The full details of such mesoscale modeling approaches are beyond the scope of this paper, but below we provide a brief overview of the treatments applied here. Figure 9 shows a 2D meshed representative volume element (RVE) reconstructed from the segmented microstructures provided by the microCT. Here, the segmented image is sieved to remove segmented particles containing fewer than 5 pixels. Contour detection algorithms based on Python’s OpenCV package [37] were used to convert the segmented HMX grains into a meshable geometry. A finite element mesh of the RVE was generated using Sandia National Laboratories’ CUBIT application [38]. The prescribed meshing routines produce a highly ordered quadrilateral mesh with refined detail around the HMX grain boundaries as seen in Figure 9. A thin layer of cohesive elements placed between regions of HMX and HTPB enable simulation of the progressive debonding and degradation of the binder-to-crystal interfaces. 

Without full characterization of the binder properties, hyperelastic parameters for HTPB as reported by Niu et al. [39] are used as a baseline in these simulations. Additionally, preliminary studies by Prakash et al. [40] and ongoing parameterization efforts suggest that a rate dependent plastic behavior is also applicable to the mechanical behavior of HTPB. Delamination behavior between the crystals and binder is modeled using a bi-linear traction-separation law to govern the previously described layer of cohesive elements. The cohesive model progressively softens the interface stiffness to zero stiffness after reaching a critical value of stress. Although Hu et al. modeled HTPB bonded ammonium perchlorate [33], their parameters for the interface model are utilized as a starting point. The 3D orientation of each HMX crystal is randomly selected from a uniform orientation distribution and assigned single crystal anisotropic elastic constants referenced from Hooks et al. [41]. Doubly periodic boundary conditions for the 2D RVE are specified following the approach developed by van der Sluis et al. [42] and the implementation scheme of Luscher et al. [43].

**Figure 10:**
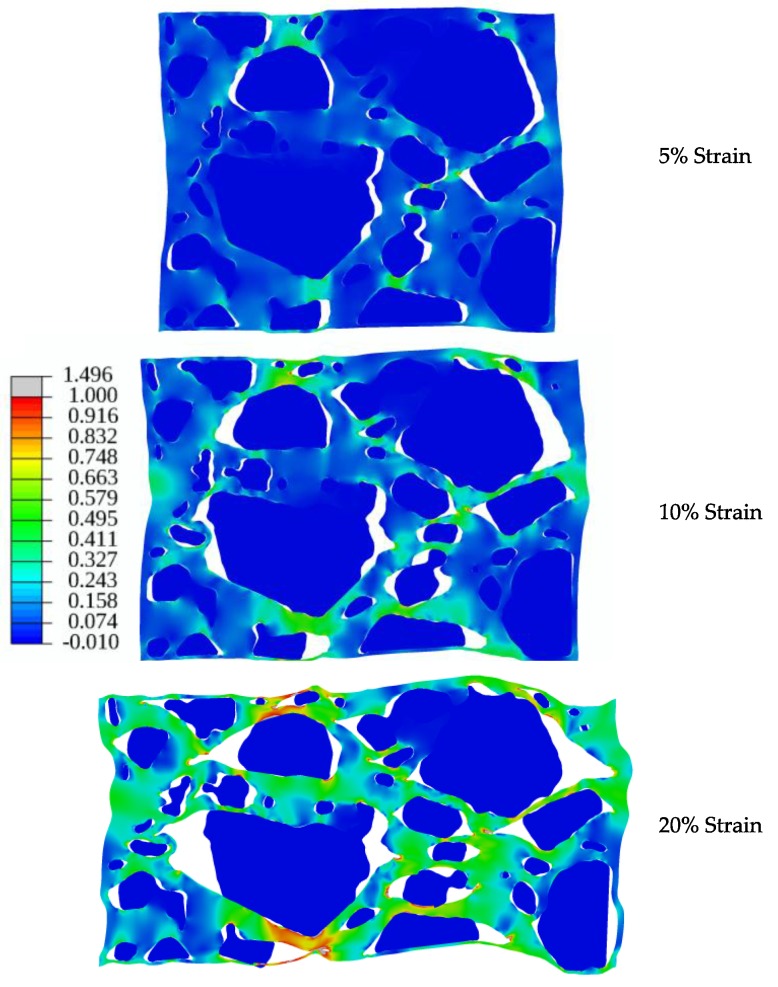
Maximum principal strain during macroscale compression of the meshed sample in Figure 9, following the general delamination behavior observed with the experimental sample **CF75H**. Failure of the crystal/binder interfaces occurs in isolated regions at first (5% strain). More surfaces are apparent at 10% strain with continued void growth at 20% strain.

To illustrate our modeling approach, a simulation utilizing a 198 *×* 182 pixel 2D section of the **CF75L** sample geometry in Figure 4 was compressed to approximately 20% strain. Figure 10 shows the progression of crystal-binder delamination at 5%, 10%, and 20%. These preliminary results exhibit qualitatively similar behavior to the high isonate formulation experiments with an HTPB binder shown in Figure 4 (**CF75H**).

**Figure 11:**
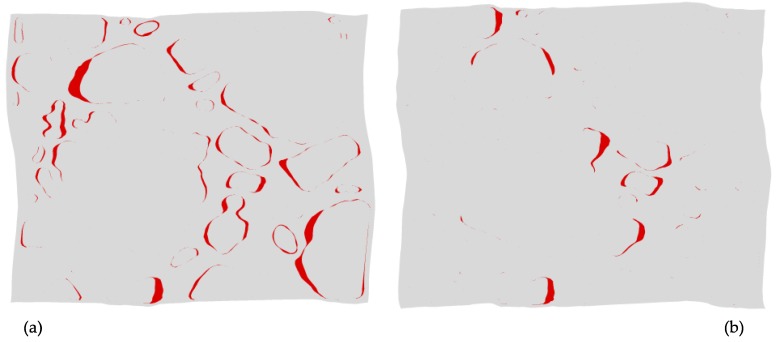
Void growth patterns at 5% macroscale strain using a polycrystalline mesh identical to those depicted in Figure 9 and Figure 10 for material parameters mimicking (**a**) sample **CF75H** and (**b**) sample **CF75L**. The red fill indicates the region of void growth associated with crystal-binder delamination.

Increasing the compliance of the HTPB binder to mimic the material behavior of the **CF75L** sample yielded familiar qualitative differences in delamination behavior between the **CF75H** and **CF75L** experimental samples. Figure 11 below shows a simple comparison of the simulated state of delamination at 5% macroscale strain for the same mesh displayed in Figure 9 and Figure 10, but with material parameters mimicking samples **CF75H** (Figure 11a) and **CF75L** (Figure 11b). Delamination at the crystal-binder interfaces are markedly less numerous for the analogous **CF75L** simulated sample than the **CF75H** simulated sample using the same mesh: similar to the comparative experimental results shown in Figure 4 and Figure 5.

Ongoing model development seeks to apply more specific characterization of the binder and interface parameters. In addition to altering the compliance of the HTPB binder, parameters which control plastic hardening of the binder and adhesion between the crystals and binder are also parameters which affect both the interface and macro-scale mechanical behavior. Simulations on increasing spatial scales will be useful for determining an appropriate RVE at which macroscale behaviors are adequately approximated. Insights gained from 2D simulation will help streamline parameterization of full 3D models using real geometric representations of PBX materials. Furthermore, accurate simulation of the characterized microstructure of the materials presented here will enable new simulations of quasistatic and dynamic mechanical deformation of PBX 9501 and other relevant materials. This current study is identifying a range of deformation and fracture responses which depend on several thermomechanical properties such as stiffness and crystal-binder adhesion. It is likely that other PBX materials fall into this spectrum of possible responses, enabling us to conduct simulations and validation experiments without needing to perform full microstructural characterization of every single sample.

## 4. Conclusions

The ability to characterize the effects of compression and damage in PBXs is integral to understanding explosives systems in general. To our knowledge, this is the first 3D spatially-resolved characterization of a PBX microstructure during deformation. A series of PBXs using HMX and lower-density binder systems which were conducive to X-ray imaging were prepared, including HTPB cured with isonate, and GAP cured with a commercial blend of acrylic monomers/oligomers. Critically, pronounced changes in the stiffness of the HTPB-based binder were found with very small adjustments in the level of isonate (0.53%–1.02%). Correspondingly, a range of deformation and fracture responses that depended on binder properties such as stiffness and crystal-binder adhesion were identified. Using micro-CT imaging during mechanical compression, we observed ductile flow with no fracture, crystal-binder delamination, intergranular cracking (where various delaminations link up to form a crack), and transgranular fracture. Software-based volume rendering of the three-dimensional microstructure was used to create a 2D mesh as a starting point for mesoscale modeling, which exhibited qualitatively similar delamination of the binder and crystals that is observed experimentally. Altering the compliance of the binder in the model to emulate the experiment resulted in void distributions that qualitatively matched the image results. Concurrent work involves tracking individual crystal flow pathways during compression. It is likely that other PBX materials fall into this spectrum of possible responses, enabling us to conduct simulations and validation experiments without needing to perform full microstructural characterization of every single sample. Future work using synchrotron tomography could provide even higher resolution via simultaneous imaging during deformation.

## Figures and Tables

**Table 1:** HMX formulations prepared and tested.

Formulation	% HMX ^1^	% HTPB/DOA/Isonate ^2^	% GAP/AMO	Description
CF75L	88.0	5.40/5.40/0.53	0/0	Low Isonate, Spongy consistency
CF75H	87.6	5.37/5.37/1.02	0/0	High Isonate, Hard cure
CF75-AMO1	88.0	0/0/0	3.0/9.0	Cured with acrylic monomers/oligomers, Hard cure
CF75-AMO2	88.0	0/0/0	6.0/6.0	Cured with acrylic monomers/oligomers, Hard cure

**^1^** HMX crystals are a mixture of 75/25 coarse/fine. **^2^** HMX, HTPB, and DOA were dried in a vacuum oven overnight before use.

**Table 2:** Volume and mass percent for HMX-HTPB samples taken from the microCT images. ^1^

Formulation	% HMX Volume	% Binder Volume	% Void Volume	% HMX Mass	% Binder Mass
**CF75L**	57.9	41.6	0.5	74.4	25.6
**CF75H**	55.9	43.6	0.5	73	27.0

**^1^** Mass calculations assume a crystal and binder density of 1.905 g/cm^3^ and 0.910 g/cm^3^, respectively.
